# Enhanced brain susceptibility to negative stimuli in adolescents: ERP evidences

**DOI:** 10.3389/fnbeh.2015.00098

**Published:** 2015-04-28

**Authors:** Jiajin Yuan, Enxia Ju, Xianxin Meng, Xuhai Chen, Siyu Zhu, Jiemin Yang, Hong Li

**Affiliations:** ^1^Key Laboratory of Cognition and Personality of Ministry of Education, Faculty of Psychology, Southwest UniversityChongqing, China; ^2^Key Laboratory of Behavior and Cognitive Psychology in Shaanxi Province, School of Psychology, Shaanxi Normal UniversityXi'an, China; ^3^School of Education, Nanyang Normal CollegeNanyang, China; ^4^Postdoctoral research station of mathematics, Southwest UniversityChongqing, China; ^5^Research Centre for Brain Function and Psychological Science, Shenzhen UniversityShenzhen, China

**Keywords:** Event-Related Potentials (ERPs), adolescence, emotion intensity, negative pictures, emotional susceptibility

## Abstract

**Background:** Previous studies investigated neural substrates of emotional face processing in adolescents and its comparison with adults. As emotional faces elicit more of emotional expression recognition rather than direct emotional responding, it remains undetermined how adolescents are different from adults in brain susceptibility to emotionally stressful stimuli.

**Methods:** Event-Related Potentials (ERPs) were recorded for highly negative (HN), moderately negative (MN), and neutral pictures in 20 adolescents and 20 adults while subjects performed a standard/deviant distinction task by pressing different keys, irrespective of the emotionality of deviant stimuli.

**Results:** Adolescents exhibited more negative amplitudes for HN vs. neutral pictures in N1 (100–150 ms), P2 (130–190 ms), N2 (210–290 ms), and P3 (360–440 ms) components. In addition, adolescents showed more negative amplitudes for MN compared to neutral pictures in N1, P2, and N2 components. By contrast, adults exhibited significant emotion effects for HN stimuli in N2 and P3 amplitudes but not in N1 and P2 amplitudes, and they did not exhibit a significant emotion effect for MN stimuli at all these components. In the 210–290 ms time interval, the emotion effect for HN stimuli was significant across frontal and central regions in adolescents, while this emotion effect was noticeable only in the central region for adults.

**Conclusions:** Adolescents are more emotionally sensitive to negative stimuli compared to adults, regardless of the emotional intensity of the stimuli, possibly due to the immature prefrontal control system over the limbic emotional inputs during adolescence.

## Introduction

Adolescence is a “stormy” developmental period that is linked with emotional instability, risky behaviors, and affective disturbances (Spear, 2000; Ernst et al., 2006; Dahl and Gunnar, 2009; Somerville et al., 2010). It has been indicated that the development of the brains' function of processing emotional stimuli lasts into 18 years old, or even to young adulthood (Levin et al., 1991). Considerable research indicates that the higher order function of prefrontal cortex, such as self-regulation, is sensitive to changes of age through adolescence to young adulthood (Luna et al., 2001; Somerville et al., 2010), and that prefrontal modulation of subcortical inputs is still immature during adolescence (Lewis et al., 2006; Hare et al., 2008). It was suggested that the immature orbital and medial prefrontal cortices are implicated in aggression and socially inappropriate behaviors in adolescents (Lewis et al., 2006; Carretié et al., 2009).

In addition, many studies using behavioral and neuroimaging measures unraveled differences between adolescents and adults during processing emotional facial expressions (Monk et al., 2003; Yurgelun-Todd and Killgore, 2006; Hare et al., 2008; Passarotti et al., 2009), a sort of emotional materials considered to elicit more of emotion recognition (Wild et al., 2001; Britton et al., 2006; Proverbio et al., 2009). For example, Yurgelun-Todd and Killgore (2006) reported that age was positively correlated with greater prefrontal cortex activation during perception of fearful faces. Using fMRI technique and an emotional go/no-go paradigm, Hare et al. (2008) observed slower behavioral response and less prefrontal relative to amygdala activity to fearful target faces in adolescents than in adults, suggesting that top-down cognitive processes are susceptible to affective interference in adolescents. More recently, Passarotti et al. (2009) compared neural bases between adolescents and adults during facial emotion processing. Adolescents, compared with adults, showed less activation in right ventrolateral prefrontal cortex and greater activation in paralimbic regions, suggesting that adolescents are more emotionally reactive relative to adults, as a result of immature prefrontal regulation of subcortical inputs. Furthermore, there was converging evidence showing that adolescents are less accurate in identifying fearful facial expressions than adults (Baird et al., 1999; Thomas et al., 2001; Yurgelun-Todd and Killgore, 2006). All these results suggest that the neural circuits and function for emotion processing are still immature during adolescence (Luna et al., 2001; Passarotti et al., 2009; Wong et al., 2009).

The human brain is equipped with an emotional negativity bias which prioritizes the processing of negative over other stimuli due to their significant adaptive values (Cacioppo and Berntson, 1994; Carretié et al., 2001; Holmes et al., 2003; Vaish et al., 2008). This bias exists stably in adults, regardless of whether the stimuli are emotionally evocative scenes (Ito et al., 1998; Huang and Luo, 2006; Yuan et al., 2007), or emotional human faces (Britton et al., 2006; Proverbio et al., 2009). Nevertheless, it is still unknown whether adolescents share this sensitivity, and what different features they may have compared to adults in response to emotionally negative scenes. Though the limbic regions of adolescents are more reactive to emotional facial expressions than those of adults, emotional facial expressions are known to induce more of facial expression recognition rather than direct emotional reaction (Wild et al., 2001; Li et al., 2008; Proverbio et al., 2009). It has yet to be determined whether adolescents are more susceptible to emotionally unpleasant scenes relative to adults, a sort of emotional materials thought to induce more direct emotional responding (Britton et al., 2006; Li et al., 2008). In particular, the emotional intensity of unpleasant scenes is important, as highly negative scenes elicit greater emotional responding (Yuan et al., 2007; Meng et al., 2009), and influence cognitive functions to a greater extent compared to mildly negative scenes (Yuan et al., 2012a). To date, it is also unclear how adolescents respond to unpleasant scenes of diverse emotion intensities, and how this emotional response varies between adolescents and adults.

Moreover, if adolescents and adults are different in susceptibility to negative scenes, this difference is not just likely to be manifested by response magnitude differences across age groups, but is also likely to be embodied by the differences in the threshold of eliciting emotional reaction to negative scenes. In this regard, it is necessary to manipulate the emotion intensity of negative pictures as highly negative (HN) and mildly negative (MN), respectively. The purpose of this manipulation is to detect the threshold differences between adolescents and adults in reaction to negative stimuli. This manipulation provides important supplementary information to the comparison of emotion-related age differences. For example, if adolescent brains are more sensitive to negative stimuli than adults, it is possible to observe emotional reactivity at a lower threshold of negative stimulation in adolescents relative to adults, in addition to the traditional methods of testing group differences in the magnitude of emotional reaction or in the speed of eliciting emotional effects. Furthermore, to better understand the age differences in sensitivity to negative scenes, it is necessary to examine timing differences in producing significant emotional effects across groups, for example, to examine whether adolescents are sooner, or later than adults in displaying emotional reactions to negative pictures. The answer to this question entails the use of a technique with high temporal resolution, such as Event-Related Potentials (ERP).

Therefore, using ERP technique, the present study investigated the differences between adolescents and adults in brain susceptibility to emotionally negative scenes, by manipulating the emotion intensity of negative scenes to be HN or MN. We used emotionally evocative scenes, instead of facial expressions, to elicit direct emotional reaction (Britton et al., 2006) instead of emotion recognition (Herba and Phillips, 2004). On the other hand, in life settings, emotional responding often occurs unpredictably and is triggered by sudden stimulations during non-emotional situations, such as accidental words from other people offend us in a conversation, as is often the case in social interaction (Yuan et al., 2007). Thus, an experimental design that does not require subjects to evaluate emotion may allow emotional responses in the laboratory setting to more closely resemble those in natural situations. Accordingly, the present study used an emotion-irrelevant distracting task which required subjects to make a non-emotional standard/deviant distinction by pressing two response keys, irrespective of the emotionality of deviant stimuli. Rather than requiring a single response for the deviants, we designed two responses to mask the true purpose of the experiment, so as to avoid a “relevance-for-task” effect that was repeatedly reported to obscure the effect of emotion on ERPs (Carretié et al., 2001). Moreover, the pictures used to elicit emotional responses in the current study were taken from the Chinese Affective Picture System (CAPS; Bai et al., 2005), because a cultural bias for the International Affective Picture System (IAPS) has been reported in Chinese subjects (Huang and Luo, 2004). As adolescents are known for immature prefrontal function of regulating subcortical inputs compared to adults, we hypothesized that adolescents may be more reactive to unpleasant pictures than adults. This may be manifested by the adolescents showing a larger emotional effect relative to adults across different processing stages, or by the significant emotion effect occurring at earlier time points in adolescents compared to adults.

Prior studies using covert tasks reported emotion effects in the early frontal N1 and P2 (Eimer et al., 2003; Holmes et al., 2003; Yuan et al., 2007; Wang et al., 2012), the frontal-central N2 (Yuan et al., 2007; Li et al., 2008), and the late parietal P3 components (Ito et al., 1998; Delplanque et al., 2004; Huang and Luo, 2006). It has been reported that N1 amplitudes were significantly different for fearful and neutral faces (Holmes et al., 2003; Rotshtein et al., 2010), while frontal P2 was more negative for both beautiful (Wang et al., 2012) and aversive pictures (Yuan et al., 2007; Meng et al., 2009) relative to neutral pictures. These early components may show enhanced emotion effect for negative stimuli in adolescents compared to adults. Additionally, a frontal-central N2, which is thought an initial index of conscious processing (Daffner et al., 2000), reflects enhanced attention orienting to infrequent stimuli (Halgren and Marinkovic, 1995; Daffner et al., 2000). This component was most pronounced when aversive pictures served as deviant stimuli (Yuan et al., 2007; Li et al., 2008), and this component has a temporal-occipital counterpart, Early Posterior Negativity that is also sensitive to emotion (EPN; Eimer et al., 2003; Schacht and Sommer, 2009; Yang et al., 2013). These attentional indexes may also reflect enhanced brain susceptibility of adolescents relative to adults. Furthermore, parietal P3 represents late conscious, cognitive processing of emotional stimuli, with access to controlled processing resources (Ito et al., 1998; Huang and Luo, 2006; Yuan et al., 2007). Because adolescents are linked with less effective frontal regulation of emotional inputs than adults, it is also likely to observe larger emotion effect in P3 amplitudes or latencies in adolescents than in adults.

## Materials and methods

### Participants

As paid volunteers, 20 adolescents aged in 13–14 years (10 boys, *M* = 13.70, *S.E*. = 0.11) and 20 adults aged 20–22 years (10 men, *M* = 20.75 years, *S.E*. = 0.47) were included in this study. All the subjects were free of brain illness history, psychiatric disorders, or medications. In addition, both samples were right-handed, with normal or corrected to normal eyesight. Also, the subjects recruited for this study were emotionally healthy, free of anxious/depressive disturbances. Adolescents and their guardians were debriefed with respect to the emotional stability of the adolescents in the past 2 weeks, by reporting whether they had the following symptoms by a yes/no fashion: irritable, uneasy, anxious, and depression. Adolescents and their guardians who reported no these symptoms were recruited for the ERP experiment. Adult subjects were screened for emotional instability by NEO-FFI-neuroticism assessment, and they scored significantly below the threshold (0) of neuroticism (*M* = −26.45; *S.E*. = 4.2; *p* < 0.001). Adolescents were tested with the Pubertal Development Scale (PDS, Wave 3; Petersen et al., 1988; Earls et al., 2002). The pubertal status of adolescents recruited for this study was determined by the following three criteria: (1), the scoring in each item of the PDS measure ranged from 2 to 4; (2), nobody reported completion of physical development (total score < 20); and (3), girls reported menarche and obvious breast growth, and boys reported deepening of voice and obvious facial hair growth (equivalent to Tanner Stage 3–4). Each adolescent had prominent features of human puberty, according to their scores in the puberty assessment (*M* ± SD:12.90 ± 2.26). The PDS and NEO-FFI questionnaires were administered in Chinese, whose validity and reliability were verified by prior studies with native Chinese subjects (Wang et al., 2010; Zhu and Chen, 2012). The study was approved by the Local Review Board for Human Participant Research and written informed consent was obtained prior to the study from all adults, and from parents/legal guardians to adolescents. The experimental procedure was in accordance with the ethical principles of the 1964 Declaration of Helsinki (World Medical Organization, 1964).

### Materials

The present study used a two-choice oddball task which consisted of four blocks of 100 trials. Each block included 70 standard and 30 deviant (grouped into three emotion conditions) pictures. All deviant pictures were taken from the Chinese adapted version of International Affective Picture System (Chinese Affective Picture System; Bai et al., 2005). A natural scene of a cup served as the frequent standard picture and 30 pictures grouped as either HN, MN, or Neutral served as the deviants. As prior findings showed that human brains are resistant to habituation to negative stimuli (Carretie et al., 2003), we repeated the presentation of all the pictures in the first block for once, to increase the number of trials for each condition. The pictures covered a variety of contents, such as highly unpleasant, mildly unpleasant, or neutral animals (e.g., snakes, bugs, or eagles), natural scenes (e.g., fire disaster, flood, clouds), and human activity (e.g., homicide, violence, or sports), but did not include single faces. The stimulus presentation was randomized across conditions.

**Figure 1:**
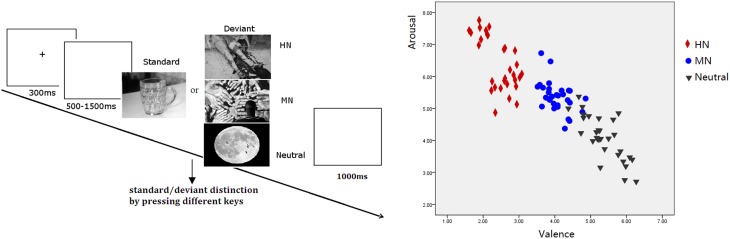
**Left:** Schematic illustration of the behavioral procedure and the picture examples; **Right:** The scatterplot for the valence and arousal for HN, MN, and Neutral pictures.

The validity of the three picture sets to serve as highly negative, mildly negative, and neutral stimuli was verified by our prior study (Yuan et al., 2012a). The stimulus reassessment with another sample of young adults (*n* = 31) showed that the three sets of deviant pictures differed significantly in valence from one another [*F*_(2, 87)_ = 357.9, *p* < 0.001]. Specifically, HN pictures were more unpleasant than MN pictures (*p* < 0.001) which, in turn, were unpleasant compared with the Neutral pictures (*p* < 0.001). Also, the three sets of pictures differed significantly in arousal [*F*_(2, 87)_ = 88.13, *p* < 0.001]. HN pictures were more arousing relative to MN pictures (*p* < 0.001) which, again, possess greater arousal than Neutral pictures (*p* < 0.001; see Figure 1). Contrast and luminance levels of the pictures were also controlled. All the pictures were identical in size and resolution (15 × 10 cm, 100 pixels per inch).

### Procedure

Subjects were seated in front of a monitor, about 150 cm from the screen, with the horizontal and vertical visual angles below 6°. To prevent fatigue, participants were allowed to take a 2-min break after each block. Stimuli were presented using E-Prime version 1.1 (Psychological Software Tools, Pittsburgh, PA). In each trial (see Figure 1), a 300 ms fixation cross was presented, which was followed by a blank screen whose duration varied randomly for 500–1500 ms. Then, a stimulus picture appeared on the screen. Subjects were instructed to press the “F” key on the keyboard with their left index finger as accurately and quickly as possible if the standard picture appeared, and to press the “J” key with their right index finger if the deviant picture appeared. The stimulus picture was terminated by a key pressing, or was terminated when it elapsed for 1000 ms. Therefore; each subject was informed that their responses must be made under 1000 ms. Each response was followed by 1000 ms of a blank screen. A practice session with 10 trials was used before the experiment to familiarize subjects with the procedure. The standard picture in the practice was the same as that in the experiment whereas the deviants for practice were neutral pictures that were not selected for the experiment. All subjects achieved 100% accuracy in practice trials.

### Electrophysiological recording and data reduction

The EEG was recorded from 64 scalp sites using tin electrodes mounted in an elastic cap (Brain Product, Munchen, Germany), with the average references on the left and right mastoids for offline ERP computation (average mastoid reference; Luck, 2005) and a ground electrode on the medial frontal aspect. The vertical electrooculograms (EOGs) were recorded supra- and infra-orbitally at the left eye. The horizontal EOG was recorded from the left vs. right orbital rim. The EEG and EOG were amplified using a recording bandpass of 0.01–100 Hz (FIR filter) at a sampling rate of 500 Hz. Data acquisition was not started until all impedance values were below 5 kΩ.

Averaging of ERPs was computed off-line using the Vision Analyzer software developed by the Brain Products Company (Munich, Germany). EEG was band-pass filtered from 0.1 to 24 Hz for offline analysis. EOG artifacts (blinks and eye movements) were corrected using the eye movement correction algorithm recommended by Gratton et al. (1983). Artifact-free EEG segments to trials with correct responses were averaged separately for each condition in adolescent and adult samples, respectively. The averaging epoch was 1000 ms, including a 200 ms pre-stimulus baseline. Trials with EOG artifacts (mean EOG voltage exceeding ±100 μV) and those contaminated with artifacts due to amplifier clipping, peak-to-peak deflection exceeding ±100 μV were excluded from averaging. The rejected trials were rare. There were 36.85 trials for HN, 36.40 trials for MN, and 35.85 trials for Neutral condition in adults, and there were 37.55 trials for HN, 37.25 trials for MN, and 38.05 trials for Neutral trials in adolescents. The number of trials used for ERP averaging was similar across the three conditions [*F*_(2, 76)_ = 0.42, ns], and the two samples [*F*_(1, 38)_ = 0.84, ns].

### Statistical analyses

**Figure 2:**
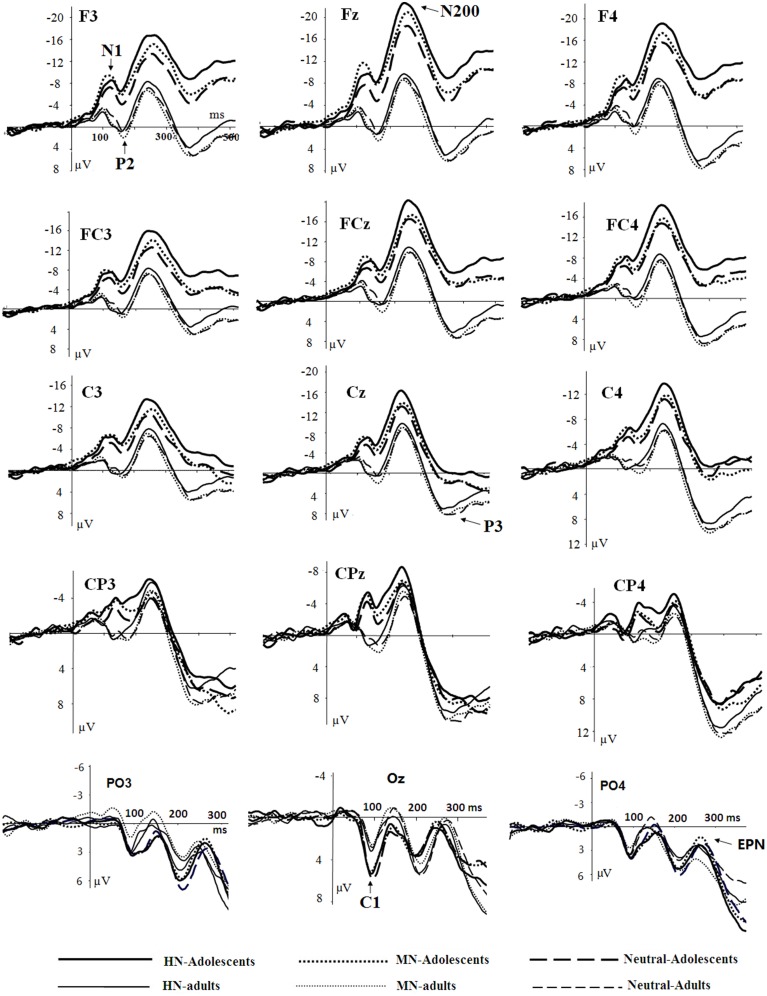
**Averaged ERPs elicited by HN (solid lines), MN (dotted lines), Neutral (dashed lines) stimuli in adolescents (bold lines) and adults (thin lines) at F3, Fz, F4 (frontal); FC3, FCz, FC4 (frontocentral); C3, Cz, C4 (central); CP3, CPz, CP4 (centroparietal), and PO3, Oz, PO4 (parietal-occipital) sites**.

**Figure 3:**
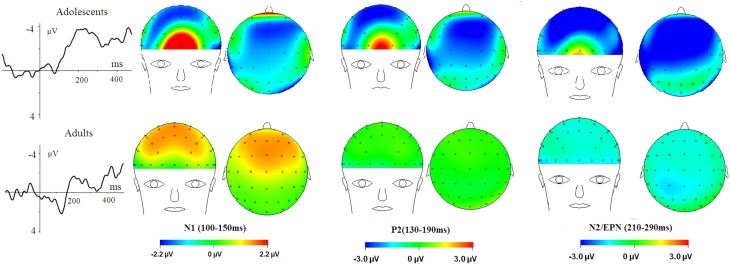
**HN–Neutral difference ERPs at FCz, and the topographical distributions of the voltage amplitudes of HN–Neutral difference ERPs in N1 (100–150 ms), P2(130–190 ms), and N2/EPN(210–290 ms) time interval in adolescents (top panel) and adults (bottom panel)**.

The averaged ERPs and topographical maps (see Figures 2, 3) showed that the amplitude differences across the three conditions started at about 100 ms post stimulus. Prominent N1 (100–150 ms), P2 (130–190 ms), and N2 (210–290 ms) components were elicited at the central and frontal sites, consistent with the scalp distributions reported by abundant ERP studies using affective pictures (Carretié et al., 2001; Huang and Luo, 2006; Yuan et al., 2007). Thus, we selected the following nine electrodes for analyses of the N1, P2, and N2 components: Frontal (F3, Fz, F4), Frontal-central (FC3, FCz, and FC4), Central (C3, Cz, C4) sites. In addition, there was a prominent P3 (360–440 ms) whose amplitudes were distributed broadly across both anterior and posterior regions (Figure 2). Because P3 had no obvious peaks at central and more posterior sites, we measured the mean amplitudes of P3 (360–440 ms) at the following 12 sites: F3, Fz, F4 (frontal), FC3, FCz, C3 (frontocentral), C3, Cz, C4 (central), and centroparietal (CP3, CPz, and CP4) sites. Accordingly, a repeated measures Analysis of Variance (ANOVA) was conducted on the peak amplitudes (defined by mean amplitudes ± 4 ms around the peak) and peak latencies of N1, P2, and N2; and the mean amplitudes of P3 component, with Emotion (HN, MN, and Neutral), frontality [three levels (F, FC, C) for N1, P2, and N2; four levels (F, FC, C, and CP) for P3] and laterality (three levels: left, midline, and right) as repeated factors while age (adolescents, adults) as a between-subjects factor. Moreover, as prior studies indicated that attention bias for affective stimuli is also reflected by an EPN in bilateral parietooccipital sites (Schupp et al., 2003; Schacht and Sommer, 2009), we analyzed the mean amplitudes of EPN (210–290 ms) at the following sites: P3/P4, PO3/PO4, P5/P6, and O1/O2; to test the age-related emotion effects at posterior scalp region. A repeated measures ANOVA was conducted, with Emotion (three levels), Laterality (left, right) as within-subject factor and age as a between-subjects factor. Lastly, occipital C1 (50–100 ms) was measured and analyzed at Oz, O1, O2, POz, PO3, and PO4, to determine whether the early visual encoding was modulated by emotion and age factors. The degrees of freedom of the F-ratio were corrected for violation of spherical assumption according to the Greenhouse–Geisser method. Bonferroni–Holm method was used for *post hoc* comparisons if significant main or interaction effects were found.

## Results

### Behavioral data

Response accuracy (ACC) reached a ceiling effect, regardless of stimulus category and group manipulations (Macc = 98.5%). As our pre-test showed violation of normal distribution in the RT data of some subjects, we used trimmed mean (the top and bottom 10% trimmed while the rest 80% for averaging) for a repeated measures ANOVA with group (two levels: adolescents and adults) × Emotion (three levels: HN, MN, and Neutral) as factors. The results showed neither significant main effects of age [*F*_(1, 38)_ = 0.08, ns] and emotion [*F*_(2, 76)_ = 1.60; *p* = 0.21], nor a significant age by emotion interaction [*F*_(2, 76)_ = 1.85; *p* = 0.16; see Supplementary Material 1].

### ERP data

#### Occipital C1 (60–100 ms)

There was no significant effect detected in the analysis of C1 latencies. The analysis of C1 amplitudes showed a significant main effect of age [*F*_(1, 38)_ = 23.58, *p* < 0.001; η^2^_*p*_ = 0.38], with adolescents exhibiting larger amplitudes compared to adults (see Figure 2). In addition, there was a significant main effect of electrode location [*F*_(5, 190)_ = 9.31, *p* < 0.001; η^2^_*p*_ = 0.20]. The amplitudes were larger at Oz compared to other sites (*p_s_* < 0.001). No other significant effects were detected in the analysis of this component.

#### Anterior N1 (100–150 ms)

The analysis of N1 amplitudes showed a significant main effect of frontality [*F*_(2, 76)_ = 32.19, *p* < 0.001; η^2^_*p*_ = 0.46], with the amplitudes more pronounced at frontal in comparison with central (*p* < 0.001) and centrofrontal (*p* < 0.001) regions. In addition, we observed a significant main effect of age [*F*_(1, 76)_ = 14.54, *p* < 0.001; η^2^_*p*_ = 0.28], and a significant age by emotion interaction [*F*_(2, 76)_ = 7.57, *p* < 0.001; η^2^_*p*_ = 0.17]. The breakdown of this interaction showed a significant emotion effect in adolescents [*F*_(2, 38)_ = 6.79, *p* = 0.003; η^2^_*p*_ = 0.26] but not in adults [*F*_(2, 38)_ = 1.71, *p* = 0.20]. Adolescents exhibited larger amplitudes for HN (*p* = 0.01) and MN (*p* = 0.01) stimuli compared to neutral stimuli; while the amplitude differences between HN and MN stimuli were not significantly different (*p* = 0.68; Table 1). The analysis of N1 latencies showed significant main effects of age [*F*_(1, 38)_ = 4.14, *p* = 0.049; η^2^_*p*_ = 0.10] and laterality [*F*_(2, 76)_ = 9.72, *p* = 0.001; η^2^_*p*_ = 0.20]. Adolescents (123 ms) elicited longer peak latencies than adults (115 ms); while the right (122 ms) sites recorded longer latencies than the midline (118 ms; *p* < 0.001) and left (118 ms; *p* = 0.006) sites.

**Table 1:** **The breakdown of the age and emotion interaction by the mean amplitude values (μV) per condition and age group**.

	**Adolescents**				**Adults**			
	**N1**	**P2**	**N2_F-FC_**	**N2_C_**	**N1**	**P2**	**N2_F-FC_**	**N2_C_**
HN	−9.78	−4.73	−20.81	−16.01	−3.62	2.50	−10.14	−9.42
MN	−10.03	−3.97	−18.57	−14.12	−3.98	3.30	−8.81	−8.26
Neutral	−7.99	−2.44	−16.83	−12.98	−4.58	2.18	−9.11	−8.11

#### Anterior P2 (130–190 ms)

Central sites recorded larger amplitudes than frontal sites, shown by a significant main effect of frontality [*F*_(2, 76)_ = 4.40, *p* = 0.037; η^2^_*p*_ = 0.10]. Adults exhibited larger amplitudes compared to adolescents [*F*_(1, 38)_ = 15.18, *p* < 0.001; η^2^_*p*_ = 0.29]. There was a significant age by emotion interaction on the P2 amplitudes [*F*_(2, 76)_ = 4.99, *p* = 0.01; η^2^_*p*_ = 0.12].

To break down this interaction, we analyzed the emotion effect in adults and adolescents, respectively. The results showed a significant emotion effect in adolescents [*F*_(2, 38)_ = 4.84, *p* = 0.017; η^2^_*p*_ = 0.20], with the amplitudes smaller for HN (*p* = 0.032) and MN (*p* = 0.039) compared to neutral stimuli (see Table 1 and Figure 3). In contrast, the emotion effect was not significant in adults [*F*_(2, 38)_ = 1.85, *p* = 0.18]. The analysis of the latencies showed no other effects except for a significant frontality, laterality and emotion interaction [*F*(8, 304) = 3.24; *p* = 0.009; η^2^_*p*_ = 0.08]. The breakdown of this interaction showed shorter peak latencies during HN compared to MN and neutral conditions at frontal, frontocentral, and right-central [*F*_(2, 76)min_ = 5.26, *p*_max_ = 0.009] but not at midline and left central sites [*F*_(2, 76)max_ = 2.47, *p* > 0.09].

#### Anterior N2 (210–290 ms)

Frontal sites (−14.41 μV) elicited larger amplitudes than central sites (−11.49 μV) and this effect was most pronounced at the midline location, indicated by a significant frontality effect [*F*_(2, 76)_ = 22.52, *p* < 0.001; η^2^_*p*_ = 0.37] and a frontality and laterality interaction [*F*_(4, 152)_ = 3.80, *p* = 0.008; η^2^_*p*_ = 0.09]. In addition, adolescents exhibited larger amplitudes than adults [*F*_(1, 38)_ = 13.29, *p* = 0.001; η^2^_*p*_ = 0.26]. This effect was more pronounced at frontal (−19.60 μV vs. −9.22 μV; *p* < 0.001) relative to central sites (−14.37 vs. −8.60 μV; *p* = 0.01), indicated by a significant frontality and age interaction [*F*_(2, 76)_ = 12.94, *p* < 0.001; η^2^_*p*_ = 0.25]. More importantly, there was a significant main effect of emotion [*F*_(2, 76)_ = 12.91, *p* < 0.001; η^2^_*p*_ = 0.25], and a significant emotion, age and frontality interaction [*F*_(4, 152)_ = 3.72, *p* = 0.02; η^2^_*p*_ = 0.09].

**Figure 4:**
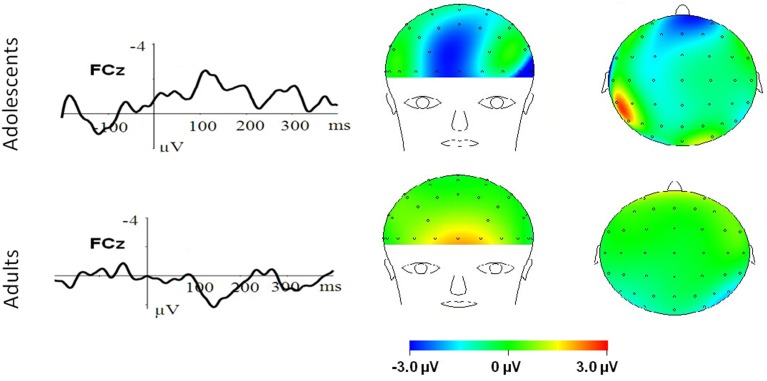
**MN–Neutral difference ERPs at FCz, and the topographical distributions of the voltage amplitudes of MN–Neutral difference ERPs in the 210–290 ms time interval in adolescents (top panel) and adults (bottom panel)**.

**Figure 5:**
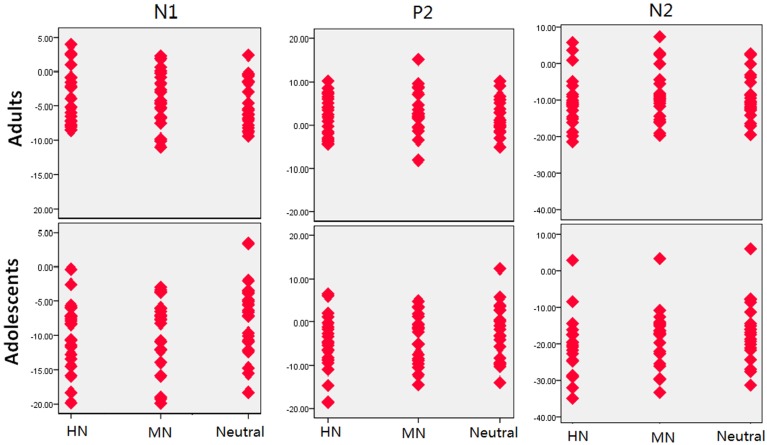
**The scatterplot for the breakdown of the age and emotion interaction in the amplitudes of each component**.

To break down this Three-Way interaction, we tested the emotion and age interaction at frontal, frontocentral, and central regions, respectively. The results showed a significant Age by emotion interaction at frontal [*F*_(2, 76)_ = 5.15, *p* = 0.009; η^2^_*p*_ = 0.12], frontocentral [*F*_(2, 76)_ = 3.66, *p* = 0.03; η^2^_*p*_ = 0.09], but not in the central region [*F*_(2, 76)_ = 1.60, *p* = 0.20]. Therefore, we decomposed the emotion by age interaction at frontal and frontocentral sites. The simple effect analysis at this region showed a significant emotion effect in adolescents [*F*_(2, 38)_ = 11.69, *p* < 0.001; η^2^_*p*_ = 0.38; Figures 3–5]. Adolescents showed larger amplitudes during HN relative to MN (*p* = 0.014) and neutral (*p* < 0.001) conditions. Also, adolescents showed more pronounced frontal amplitudes during MN compared to neutral conditions (*p* = 0.026). By contrast, the emotion effect was statistically non-significant in adults [*F*_(2, 76)_ = 2.99, *p* = 0.064]. Additionally, the lack of an age and emotion interaction in central sites was due to larger amplitudes during HN, but not MN, compared to Neutral conditions in both adolescents [*F*_(2, 38)_ = 7.41, *p* = 0.002; η^2^_*p*_ = 0.28] and adults [*F*_(2, 38)_ = 3.49, *p* = 0.042; η^2^_*p*_ = 0.16; Figures 3, 4].

As previous studies showed that sex modulated emotional effects in N200 component (Campanella et al., 2004; Yuan et al., 2009), we further conducted an analysis of covariance (ANCOVA) with sex as a covariate, to test whether the above results were reliable after controlling the possible impact of sex. The results showed a significant emotion and Age interaction [*F*_(2, 74)_ = 3.56, *p* = 0.034; η^2^_*p*_ = 0.09], and an emotion, Age, and frontality interaction [*F*_(4, 150)_ = 3.70, *p* = 0.021; η^2^_*p*_ = 0.09] after controlling the potential influence of sex. The analysis of N2 latencies showed no other effects except for a significant frontality by Age interaction [*F*_(2, 76)_ = 6.98, *p* = 0.007; η^2^_*p*_ = 0.16]. Adolescents showed a trend of prolonged peak latencies relative to adults in frontal (*p* = 0.06), but not other regions (all *p* > 0.20).

#### EPN (210–290 ms)

The analysis of EPN amplitudes showed no significant main effect of emotion [*F*_(2, 76)_ = 2.65, *p* = 0.081], or significant emotion by age interaction[*F*_(2, 76)_ = 1.97, *p* = 0.15]. Thus, the age effect in N200 responding for negative pictures was localized to frontal and frontocentral regions.

#### P3 (330–440 ms)

The amplitudes were larger at parietal compared to central and frontal sites [*F*_(3, 114)_ = 104.24, *p* < 0.001; η^2^_*p*_ = 0.73], and were larger at midline and right compared to left sites [*F*_(2, 76)_ = 10.49, *p* < 0.001; η^2^_*p*_ = 0.22]. Adults elicited larger amplitudes than adolescents in frontal, frontocentral and central [*F*_(1, 38)min_ = 6.28, *p*_max_ = 0.017] but not in centroparietal [*F*_(1, 38)_ < 1, ns] regions, shown by a significant age and frontality interaction [*F*_(3, 114)_ = 28.57, *p* < 0.001; η^2^_*p*_ = 0.43]. There was a significant emotion and frontality interaction [*F*_(6, 228)_ = 6.28, *p* = 0.001; η^2^_*p*_ = 0.14], with a significant emotion effect observed at frontal, frontocentral, and central [*F*_(2, 76)min_ = 3.77, *p*_max_ = 0.032] but not centroparietal [*F*_(2, 76)_ = 1.79, *p* = 0.18] regions. In central-to-frontal regions, HN stimuli elicited smaller amplitudes than neutral stimuli [*F*_(1, 38)_ = 9.56, *p* = 0.008; η^2^_*p*_ = 0.20], regardless of age; while the amplitude differences for MN and neutral stimuli were not significant [*F*_(1, 38)_ < 0.1, ns]. The age and emotion interaction was not significant [*F*_(2, 76)_ < 0.3, ns].

#### The timing of the emotion effects in different samples

Summarizing the above results leaves an impression that the emotional ERP effects for HN stimuli occurred earlier in adolescents (from N1) relative to adults (from N2). To verify this impression, we directly tested the timing effect by conducting an additional ANOVA with emotion (two levels: HN and Neutral), timing (three levels: N1, P2, and N2) as repeated factors and Age as a between-subject factor. We focused this analysis on the central sites (C3, Cz, and C4) because both samples showed emotion effects in this region. The results showed a significant main effect of emotion [*F*_(1, 38)_ = 12.70, *p* = 0.001; η^2^_*p*_ = 0.25] and a significant interaction effect between age and emotion [*F*_(1, 38)_ = 10.81, *p* = 0.002; η^2^_*p*_ = 0.22] which, in turn, interacted significantly with timing [*F*_(2, 76)_ = 5.75, *p* = 0.008; η^2^_*p*_ = 0.13]. The simple effect analysis showed a significant timing and emotion interaction in adults [*F*_(2, 38)_ = 8.07, *p* = 0.002; η^2^_*p*_ = 0.30] but not in adolescents [*F*_(2, 38)_ = 1.18, *p* = 0.30]. Adults showed a significant emotion effect for HN stimuli in N2 [*F*_(1, 19)_ = 5.32, *p* = 0.032; η^2^_*p*_ = 0.22] but not in N1 [*F*_(1, 19)_ = 3.02, *p* = 0.10] and P2 [*F*_(1, 19)_ = 0.31, ns] amplitudes. By contrast, adolescents showed a significant emotion effect for HN stimuli in both N1 [*F*_(1, 19)_ = 7.72, *p* = 0.012; η^2^_*p*_ = 0.29], P2 [*F*_(1, 19)_ = 6.06, *p* = 0.024; η^2^_*p*_ = 0.24], and N2 [*F*_(1, 19)_ = 16.00, *p* = 0.001; η^2^_*p*_ = 0.46] amplitudes. These results verified that the emotion effect occurred earlier in adolescents compared to adults. Also, to further test the reliability of the faster timing of adolescents vs. adults to react to negative stimuli, we continuously measured and analyzed the mean amplitudes of each 50 ms epoch from stimulus onset to 500 ms post stimulus. The results further supported the above timing effect (see Supplementary Material 2).

## Discussion

The present study observed significant age by emotion interaction effects in the amplitudes of N1, P2, and N2 amplitudes. Adolescents exhibited significant emotion effects for both HN and MN stimuli in N1 (100–150 ms), P2(130–190 ms), and N2 (210–290 ms) components. By contrast, adults exhibited no other emotion effects in these components except for enhanced N2 amplitudes for HN compared to neutral stimuli in central sites. These results suggest that adolescents are equipped with enhanced brain susceptibility to emotionally negative scenes compared to adults.

**Figure 6:**
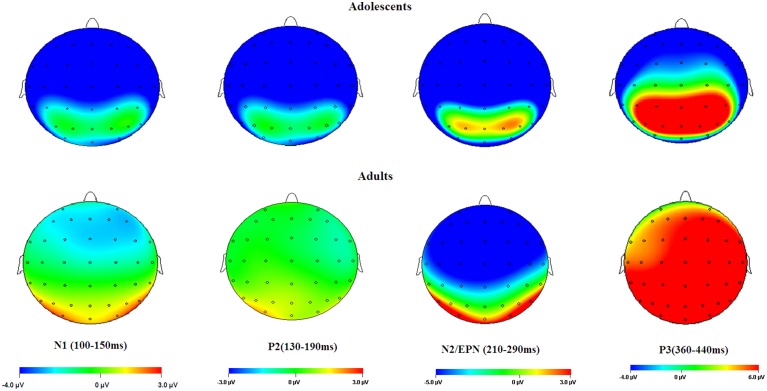
**Topographical maps of the voltage amplitudes elicited by neutral stimuli in the N1 (100–150 ms), P2 (130–190 ms), N2/EPN (210–290 ms), and P3 (360–440 ms) time intervals**.

Overall, adolescents showed greater C1 amplitudes, and also more pronounced negative deflections across N1, P2, N2, and P3 components in comparison with adults, regardless of whether the stimulus is emotional or neutral (see Figures 2, 6). Also, adolescents showed significantly delayed peak latencies in N1 and a trend of delayed latencies in N2 component compared to adults. The early components like C1 and N1 were thought to reflect basic visual processing (Pourtois et al., 2004). There is a recent review showing that the basic visual functions of children, for example, visual acuity and spatial vision, are not fully mature until mid-teenage (Leat et al., 2009). Thus, the adolescent subjects in our study (age 13–14 years) may be not fully mature in basic visual functions, which in turn led to larger C1 and N1 amplitudes during early visual processing. Also, these findings were consistent with a number of child development studies showing reduced ERP amplitudes with age in multiple tasks, such as smaller frontal N2 in AX-CPT (Jonkman, 2006) and Go/nogo (Lewis et al., 2006) tasks, and smaller P1 and N170 in repetition detection tasks (Itier and Taylor, 2004; Taylor et al., 2004). This is most likely due to the more developed executive function, such as attention maintenance and focusing on task-related processing (Jonkman, 2006; Lewis et al., 2006) in adults relative to adolescents. These attention differences across age should have been more convincing, if the current study had synchronized ERP data with an eye-tracking assessment. In addition, adolescence development is linked with continued head growth and increases in skull density and thickness (Knott et al., 2004; Segalowitz et al., 2010), which may also contribute to smaller ERP amplitudes for neutral stimuli in adults relative to adolescents.

In the present study, occipital C1 activity was not influenced by emotion, suggesting that early visual encoding of the pictures was not influenced by emotional information during the current task. Prior studies have suggested that the central presentation of emotional visual stimuli may cancel out the emotion effect in C1 amplitudes, which was however reliably observed during lateral presentation of salient stimuli (Pourtois et al., 2004; Stolarova et al., 2006). The present study presented emotional pictures in the center of the screen, without manipulation of spatial locations of the pictures. This possibly explains why we did not observe emotion-related C1 modulation in the first 100 ms post stimulus.

We observed a significant age by emotion interaction on the N1 amplitudes 100–150 ms post stimulus onset. Adolescents exhibited larger negative amplitudes for HN and MN stimuli in comparison with neutral stimuli; while adults did not show significant emotion effects for both stimulus sets. Previous studies using adult samples indicated that the frontal N1 amplitudes for negative stimuli was modulated by attention focus: while N1 amplitudes were clearly differentiated between fearful and neutral faces when attention was focused on faces (Holmes et al., 2003; Pourtois et al., 2004), this emotional effect was completely eliminated when attention was diverted away from the faces (Holmes et al., 2003). Furthermore, this early emotion effect was considered to be mediated by amygdala activity (Holmes et al., 2003; Rotshtein et al., 2010). It is worth noting that we used a non-emotional distracting task that diverts subjects' attention away from the emotionality of pictures. This probably explains why we did not observe a significant emotion effect in the adult sample, consistent with the results by Holmes et al. (2003). Nevertheless, adolescents showed a significant emotion effect for both HN and MN stimuli, despite involvement in the distracting task. It was indicated that adolescence is associated with the immature prefrontal control system and the relative maturity of subcortical systems (Giedd et al., 1999; Sowell et al., 2002; Somerville et al., 2010). Consequently, the amygdala signaling should be stronger in response to salient stimuli in adolescents compared to adults, which may explain the pronounced early N1 effect for both HN and MN negative stimuli in adolescents but not in adults.

There was a P2 component peaking approximately 160 ms after stimulus onset whose activity was distributed across frontal and central sites. The analysis of the P2 amplitudes displayed a significant emotion effect for both HN and MN stimuli in adolescents but not in adults. It has been suggested that frontal P2 is linked with exogenous, stimulus-driven attention (Carretié et al., 2013), and its amplitudes are enhanced with greater attention to salient stimuli (Carretié et al., 2001). The activation of this component is considered indicative of rapid detection of salient stimulus features (Thorpe et al., 1996; Yuan et al., 2007). Therefore, this finding suggests that adolescents detect emotional negativity of HN and MN stimuli rapidly, within 200 ms post stimulus, where information processing was considered to occur automatically, inaccessible to conscious and controlled processing resources (Thorpe et al., 1996; Huang and Luo, 2006; Del Cul et al., 2007). However, adult sample did not show biased processing of negative stimuli at this early stage. These results were consistent with a recent functional MRI study showing greater paralimbic activation and enhanced emotional reactivity to emotionally salient faces in adolescents compared to adults during a non-emotional task (Passarotti et al., 2009). Also, there was recent evidence showing reduced P2 amplitudes with enhanced emotional control skills, as measured by social skills inventory (Meaux et al., 2014). In this regard, the current finding of a significant P2 amplitude effect in adolescents but not in adults also implies that adolescents may be less skillful than adults in reaction to negative stimuli. Future studies need to directly test whether adolescents are less capable of regulating unpleasant emotion compared to adults, by using an emotional regulation task.

In the 210–290 ms interval, there was a prominent N2 whose amplitudes were largest at frontal sites, fitting the archetype of the visual N2b that has been considered to represent voluntary attention orienting to deviant stimuli, with initial access to conscious processing resources (Daffner et al., 2000). In this time interval adolescents exhibited significant emotion effects for HN stimuli across frontal and central regions, while adults showed an emotion effect for HN stimuli only in the central region. This suggests that the emotion effect for HN stimuli in this time window is more robust, with broader scalp distributions in adolescents relative to adults (see Figure 3). In addition, while MN stimuli elicited a significant emotion effect at frontal sites in adolescents, adults did not exhibit any emotion effect for MN stimuli (see Figure 4). These results suggest that adolescents are more sensitive to negative scenes than adults, such that adolescents, but not adults, directed enhanced voluntary attention to MN stimuli than to neutral stimuli, though the emotional saliency of MN stimuli is compromised relative to that of HN stimuli. This result, together with relevant findings from N1 and P2 analyses, jointly suggest that adolescent brains are indeed more sensitive to negative stimuli than adults, such that adolescents elicited significant emotional reactivity across N1, P2, and N2 stages despite weak negative stimulations. These results were consistent with the timing analysis which yielded a significantly faster emotion effect in brain potentials in adolescents relative to adults. Thus, adolescents' enhanced susceptibility to negative stimuli is not only manifested by the adolescents-specific emotion effect for MN stimuli in N1, P2, and N2 components, but is also reflected by the broader scalp activations during HN stimuli in the 210–290 ms and the faster elicitation of emotion effect in adolescent relative to adult samples.

It is worth noting that the analysis of P3 amplitudes yielded a significant emotion effect while the age by emotion interaction was non-significant. HN stimuli, rather than MN stimuli, elicited smaller amplitudes than neutral stimuli in both adolescents and adults. This was in contrast with the results from N1, P2 and N2 components, where adolescents exhibited more robust emotion effects than adults. As stated above, N1 and P2 represent early sensory and perceptual processing (Holmes et al., 2003; Pourtois et al., 2004; Carretié et al., 2013); while visual-evoked N2 reflects voluntary attention orienting to salient stimuli with initial access to conscious processing resources (Daffner et al., 2000; Del Cul et al., 2007). By contrast, parietal P3 component involves elaborated cognitive processing of stimulus meanings, with full access to consciously controlled processing resources (Ito et al., 1998; Huang and Luo, 2006). Though the early perceptual processing of negative stimuli and the attention orienting to these stimuli are enhanced in adolescents than in adults, the two groups showed similar patterns of emotional effects in P3 stage. This is likely due to the recruitment of a similar top-down controlled processing across samples, i.e., similar inhibition of task-irrelevant emotional meanings to focus on the task of standard/deviant distinction (Yuan et al., 2012b; Yang et al., 2013). This assumption was supported by our behavioral data showing no significant age differences in both response time and accuracy. Also, this inhibitory explanation was supported by the less pronounced P3 amplitudes for HN compared to neutral pictures in both samples, as prior studies of cognitive control consistently showed smaller P3 amplitudes during inhibition of task-irrelevant distracting information (Liotti et al., 2000; Chen et al., 2008; Yuan et al., 2011).

Previous studies have indicated that there is a maturity imbalance between prefrontal cortex and subcortical limbic regions during adolescence. It was reported that adolescence is characterized by immature prefrontal function and relatively matured subcortical structures (Giedd et al., 1999; Sowell et al., 2002), and that higher-order prefrontal areas undergo protracted pruning of gray matter through adolescence to early adulthood (Giedd et al., 1999; Somerville et al., 2010). This may enable stronger signaling of subcortical system (e.g., amygdala) paired with weaker top-down control signaling from neocortices in adolescents than in adults, whose subcortical and cortical systems have both matured and their interactive functioning has reached a balance (Somerville et al., 2010). This most likely accounts for the robust early emotion effects for negative stimuli in adolescents but not in adults, as indicated by the N1 and P2 analysis. On the other hand, it has been established that N2 activity elicited by visual deviant stimuli is mediated by anterior cingulated cortex (ACC; Nieuwenhuis et al., 2003; Carretié et al., 2004), whose activation is positively associated with negative mood induction in healthy subjects (Mayberg et al., 1999). It has been reported that adolescents are linked with enhanced ACC activations in response to fearful faces in comparison with adults (Monk et al., 2003), and that ACC has increasing functional connectivity with amygdala during depression (Connolly et al., 2013). These evidences offer an explanation why adolescents showed more pronounced emotion effects for negative pictures in N2 amplitudes than adults, regardless of emotion intensity.

In summary, using ERP technique, the present study observed enhanced brain susceptibility to emotionally negative stimuli in adolescents compared to adults. This enhanced susceptibility was manifested by the greater emotional effects for negative stimuli in adolescents compared to adults, regardless of the valence strength, in the amplitudes of N1, P2, and N2 components. This difference is possibly due to the oversignaling of limbic system (e.g., Amygdala, ACC) as a result of less modulatory signaling from prefrontal system during adolescence compared to adulthood. These findings contribute to the understanding of adolescents' susceptibility to affective and behavioral disturbances, such as depression, anxiety disorders, and aggression (Hankin et al., 1998; Rudolph, 2002).

However, several limitations need to be overtly acknowledged. Firstly, the current study used native neuroticism scale to assess emotional stability of adults (Wang et al., 2010) but, as the validity of this scale for adolescents has not been verified, a self and parent report method was used to assess emotional stability for adolescents. Though both samples were verified emotionally stable, this approach does not allow us to directly compare emotional stability of two samples. In this regard, future studies should aim at developing a unified scale suitable for assessing emotional stability of both adults and adolescents. Secondly, the current study did not use social skills inventory (Riggio, 2003; Meaux et al., 2014) to overtly assess the endogenous trait of emotional susceptibility for the two samples, which should have reinforced our ERP findings if there were indeed higher self-reported emotional susceptibility scores in adolescents than adults. Future studies need to directly assess this trait, for examining the correlation between neurophysiologic and behavioral indexes of emotional susceptibility. Thirdly, due to the limited spatial resolution of ERP technique, the current study provided no direct evidence for oversignaling of limbic system and less activation of prefrontal system in adolescents compared to adults. Future studies need to employ a suitable technique like MEG, to directly address the neural origins of the current findings.

### Conflict of interest statement

The authors declare that the research was conducted in the absence of any commercial or financial relationships that could be construed as a potential conflict of interest.

## References

[B1] Bai L. Ma H. Huang Y. X. Luo Y. J. (2005). The development of native chinese affective picture system–a pretest in 46 college students. Chin. Mental Health J. 19, 719–722.

[B2] Baird A. A. Gruber S. A. Fein D. A. Mass L. C. Steingard R. J. Renshaw P. F.. (1999). Functional magnetic resonance imaging of facial affect recognition in children and adolescents. J. Am. Acad. Child Adolesc. Psychiatry 38, 195–199. 10.1097/00004583-199902000-00019 9951219

[B5] Britton J. C. Taylor S. F. Sudheimer K. D. Liberzon I. (2006). Facial expressions and complex IAPS pictures: common and differential networks. Neuroimage 31, 906–919. 10.1016/j.neuroimage.2005.12.050 16488159

[B6] Cacioppo J. T. Berntson G. G. (1994). Relationship between attitudes and evaluative space: a critical review, with emphasis on the separability of positive and negative substrates. Psychol. Bull. 115, 401 10.1037/0033-2909.115.3.401

[B7] Campanella S. Rossignol M. Mejias S. Joassin F. Maurage P. Debatisse D.. (2004). Human gender differences in an emotional visual oddball task: an event-related potentials study. Neurosci. Lett. 367, 14–18. 10.1016/j.neulet.2004.05.097 15308288

[B8] Carretié L. Albert J. López-Martín S. Tapia M. (2009). Negative brain: an integrative review on the neural processes activated by unpleasant stimuli. Int. J. Psychophysiol. 71, 57–63. 10.1016/j.ijpsycho.2008.07.006 18727941

[B9] Carretié L. Hinojosa J. A. Martín-Loeches M. Mercado F. Tapia M. (2004). Automatic attention to emotional stimuli: neural correlates[J]. Hum. Brain Mapp. 22, 290–299. 10.1002/hbm.20037 15202107 PMC6871850

[B10] Carretie L. Hinojosa J. A. Mercado F. (2003). Cerebral patterns of attentional habituation to emotional visual stimuli. Psychophysiology 40, 381–388. 10.1111/1469-8986.00041 12946112

[B11] Carretié L. Kessel D. Carboni A. López-Martín S. Albert J.. (2013). Exogenous attention to facial vs. non-facial emotional visual stimuli. Soc. Cogn. Affect. Neurosci. 8, 764–773. 10.1093/scan/nss068 22689218 PMC3791067

[B12] Carretié L. Mercado F. Tapia M. Hinojosa J. A. (2001). Emotion, attention, and the ‘negativity bias,’ studied through event-related potentials. Int. J. Psychophysiol. 41, 75–85. 10.1016/S0167-8760(00)00195-1 11239699

[B13] Chen A. Xu P. Wang Q. Luo Y. Yuan J. Li H.. (2008). The timing of cognitive control in partially incongruent categorization. Hum. Brain Mapp. 29, 1028–1039. 10.1002/hbm.20449 17894393 PMC6871019

[B15] Connolly C. G. Wu J. Ho T. C. Hoeft F. Wolkowitz O. Eisendrath S.. (2013). Resting-state functional connectivity of subgenual anterior cingulate cortex in depressed adolescents. Biol. Psychiatry 74, 898–907. 10.1016/j.biopsych.2013.05.036 23910949 PMC4103629

[B16] Daffner K. R. Mesulam M. Scinto L. F. Calvo V. Faust R. Holcomb P. J. (2000). An electrophysiological index of stimulus unfamiliarity. Psychophysiology 37, 737–747. 10.1111/1469-8986.3760737 11117454

[B18] Dahl R. E. Gunnar M. R. (2009). Heightened stress responsiveness and emotional reactivity duringpubertal maturation: implications for psychopathology. Dev. Psychopathol. 21, 1–6. 10.1017/S0954579409000017 19144219

[B20] Del Cul A. Baillet S. Dehaene S. (2007). Brain dynamics underlying the nonlinear threshold for access to consciousness. PLoS Biol. 5:e260. 10.1371/journal.pbio.0050260 17896866 PMC1988856

[B21] Delplanque S. Lavoie M. E. Hot P. (2004). Modulation of cognitive processing by emotional valence studied through event-related potentials in humans. Neurosci. Lett. 356, 1–4. 10.1016/j.neulet.2003.10.014 14746887

[B22] Earls F. J. Brooks-Gunn J. Raudenbush S. W. (2002). Project on Human Development in Chicago Neighborhoods (PHDCN). Ann Arbor, MI: Inter-university Consortium for Political and Social Research.

[B24] Eimer M. Holmes A. McGlone F. P. (2003). The role of spatial attention in the processing of facial expression: an ERP study of rapid brain responses to six basic emotions. Cogn. Affect. Behav. Neurosci. 3, 97–110. 10.3758/CABN.3.2.97 12943325

[B25] Ernst M. Pine D. S. Hardin M. (2006). Triadic model of the neurobiology of motivated behavior in adolescence. Psychol. Med. 36, 299–312. 10.1017/S0033291705005891 16472412 PMC2733162

[B26] Giedd J. N. Blumenthal J. Jeffries N. O. Castellanos F. X. Liu H. Zijdenbos A.. (1999). Brain development during childhood and adolescence: a longitudinal MRI study. Nat. Neurosci. 2, 861–863. 10.1038/13158 10491603

[B28] Gratton G. Coles M. G. Donchin E. (1983). A new method for off-line removal of ocular artifact. Electroencephalogr. Clin. Neurophysiol. 55, 468–484. 10.1016/0013-4694(83)90135-9 6187540

[B29] Halgren E. Marinkovic K. (1995). Neurophysiological networks integrating human emotions, in The Cognitive Neuroscience, ed Gazzaniga M. S. (Cambridge, MA: MIT Press), 1137–1151.

[B30] Hankin B. L. Abramson L. Y. Moffitt T. E. Silva P. A. McGee R. Angell K. E. (1998). Development of depression from preadolescence to young adulthood: emerging gender differences in a 10-year longitudinal study. J. Abnorm. Psychol. 107, 128. 10.1037/0021-843X.107.1.128 9505045

[B31] Hare T. A. Tottenham N. Galvan A. Voss H. U. Glover G. H. Casey B. J. (2008). Biological substrates of emotional reactivity and regulation in adolescence during an emotional go-nogo task. Biol. Psychiatry 63, 927–934. 10.1016/j.biopsych.2008.03.015 18452757 PMC2664095

[B32] Herba C. Phillips M. (2004). Annotation: development of facial expression recognition from childhood to adolescence: behavioural and neurological perspectives. J. Child Psychol. Psychiatry 45, 1185–1198. 10.1111/j.1469-7610.2004.00316.x 15335339

[B33] Holmes A. Vuilleumier P. Eimer M. (2003). The processing of emotional facial expression is gated by spatial attention: evidence from event-related brain potentials. Brain Res. Cogn. Brain Res. 16, 174–184. 10.1016/S0926-6410(02)00268-9 12668225

[B34] Huang Y.-X. Luo Y.-J. (2004). Native assessment of international affective picture system. Chin. J. Clin. Psychol. 9, 631–634. 25381023

[B35] Huang Y.-X. Luo Y.-J. (2006). Temporal course of emotional negativity bias: an ERP study. Neurosci. Lett. 398, 91–96. 10.1016/j.neulet.2005.12.074 16446031

[B36] Itier R. J. Taylor M. J. (2004). Face recognition memory and configural processing: a developmental ERP study using upright, inverted, and contrast-reversed faces. J. Cogn. Neurosci. 16, 487–502. 10.1162/089892904322926818 15072683

[B37] Ito T. A. Larsen J. T. Smith N. K. Cacioppo J. T. (1998). Negative information weighs more heavily on the brain: the negativity bias in evaluative categorizations. J. Pers. Soc. Psychol. 75, 887. 10.1037/0022-3514.75.4.887 9825526

[B38] Jonkman L. M. (2006). The development of preparation, conflict monitoring and inhibition from early childhood to young adulthood; a Go/Nogo ERP study. Brain Res. 1097, 181–193. 10.1016/j.brainres.2006.04.064 16729977

[B39] Knott P. D. Hazony D. Karafa M. Koltai P. J. (2004). High-frequency ultrasound in the measurement of pediatric craniofacial integrity. Otolaryngol. Head Neck Surg. 131, 851–855. 10.1016/j.otohns.2004.08.010 15577779

[B40] Leat J. Yadav N. K. Irving E. L. (2009). Development of visual acuity and contrast sensitivity in children. J. Optom. 2, 19–26 10.3921/joptom.2009.19

[B41] Levin H. S. Culhane K. A. Hartmann J. Evankovich K. Mattson A. J. Harward H. (1991). Developmental changes in performance on tests of purported frontal lobe functioning. Dev. Neuropsychol. 7, 377–395 10.1080/87565649109540499

[B42] Lewis M. D. Lamm C. Segalowitz S. J. Stieben J. Zelazo P. D. (2006). Neurophysiological correlates of emotion regulation in children and adolescents. J. Cogn. Neurosci. 18, 430–443. 10.1162/jocn.2006.18.3.430 16513007

[B43] Li H. Yuan J. Lin C. (2008). The neural mechanism underlying the female advantage in identifying negative emotions: an event-related potential study. Neuroimage 40, 1921–1929. 10.1016/j.neuroimage.2008.01.033 18343686

[B44] Liotti M. Woldorff M. G. Perez R. Mayberg H. S. (2000). An ERP study of the temporal course of the Stroop color-word interference effect. Neuropsychologia 38, 701–711. 10.1016/S0028-3932(99)00106-2 10689046

[B45] Luck S. J. (2005). An Introduction to the Event-related Potential Technique (Cognitive Neuroscience). Cambridge, MA: MIT Press.

[B46] Luna B. Thulborn K. R. Munoz D. P. Merriam E. P. Garver K. E. Minshew N. J.. (2001). Maturation of widely distributed brain function subserves cognitive development. Neuroimage 13, 786–793. 10.1006/nimg.2000.0743 11304075

[B47] Mayberg H. S. Liotti M. Brannan S. K. McGinnis S. Mahurin R. K. Jerabek P. A.. (1999). Reciprocal limbic-cortical function and negative mood: converging PET findings in depression and normal sadness. Am. J. Psychiatry 156, 675–682. 10327898 10.1176/ajp.156.5.675

[B48] Meaux E. Roux S. Batty M. (2014). Early visual ERPs are influenced by individual emotional skills. Soc. Cogn. Affect. Neurosci. 9, 1089–1098. 10.1093/scan/nst084 23720573 PMC4127009

[B49] Meng X. Yuan J. Li H. (2009). Automatic processing of valence differences in emotionally negative stimuli: evidence from an ERP study. Neurosci. Lett. 464, 228–232. 10.1016/j.neulet.2009.08.064 19720111

[B50] Monk C. S. McClure E. B. Nelson E. E. Zarahn E. Bilder R. M. Leibenluft E.. (2003). Adolescent immaturity in attention-related brain engagement to emotional facial expressions. Neuroimage 20, 420–428. 10.1016/S1053-8119(03)00355-0 14527602

[B52] Nieuwenhuis S. Yeung N. van den Wildenberg W. Ridderinkhof K. R. (2003). Electrophysiological correlates of anterior cingulate function in a go/no-go task: effects of response conflict and trial type frequency. Cogn. Affect. Behav. Neurosci. 3, 17–26. 10.3758/CABN.3.1.17 12822595

[B53] Passarotti A. M. Sweeney J. A. Pavuluri M. N. (2009). Neural correlates of incidental and directed facial emotion processing in adolescents and adults. Soc. Cogn. Affect. Neurosci. 4, 387–398. 10.1093/scan/nsp029 20035016 PMC2799953

[B54] Petersen A. C. Crockett L. Richards M. (1988). A self-report measure of pubertal status: reliability, validity, and initial norms. J. Youth Adolesc. 17, 117–133. 10.1007/BF01537962 24277579

[B55] Pourtois G. Grandjean D. Sander D. Vuilleumier P. (2004). Electrophysiological correlates of rapid spatial orienting towards fearful faces[J]. Cereb. Cortex 14, 619–633. 10.1093/cercor/bhh023 15054077

[B56] Proverbio A. M. Adorni R. Zani A. Trestianu L. (2009). Sex differences in the brain response to affective scenes with or without humans. Neuropsychologia 47, 2374–2388. 10.1016/j.neuropsychologia.2008.10.030 19061906

[B57] Riggio R. E. (2003). Socials Skills Manual, 2nd Edn. Menlo Park, CA: Mind Garden Inc.

[B58] Rotshtein P. Richardson M. P. Winston J. S. (2010). Amygdala damage affects event-related potentials for fearful faces at specific time windows. Hum. Brain Mapp. 31, 1089–1105. 10.1002/hbm.20921 20017134 PMC3173845

[B59] Rudolph K. D. (2002). Gender differences in emotional responses to interpersonal stress during adolescence. J. Adolesc. Health 30, 3–13. 10.1016/S1054-139X(01)00383-4 11943569

[B60] Schacht A. Sommer W. (2009). Time course and task dependence of emotion effects in word processing. Cogn. Affect. Behav. Neurosci. 9, 28–43. 10.3758/CABN.9.1.28 19246325

[B62] Schupp H. T. Markus J. Weike A. I. Hamm A. O. (2003). Emotional facilitation of sensory processing in the visual cortex. Psychol. Sci. 14, 7–13. 10.1111/1467-9280.01411 12564747

[B63] Segalowitz S. J. Santesso D. L. Jetha M. K. (2010). Electrophysiological changes during adolescence: a review. Brain Cogn. 72, 86–100. 10.1016/j.bandc.2009.10.003 19914761

[B64] Somerville L. H. Jones R. M. Casey B. (2010). A time of change: behavioral and neural correlates of adolescent sensitivity to appetitive and aversive environmental cues. Brain Cogn. 72, 124–133. 10.1016/j.bandc.2009.07.003 19695759 PMC2814936

[B66] Sowell E. R. Trauner D. A. Gamst A. Jernigan T. L. (2002). Development of cortical and subcortical brain areas in childhood and adolescence: a structural MRI study. Dev. Med. Child Neurol. 44, 4–16. 10.1017/S0012162201001591 11811649

[B67] Spear L. P. (2000). The adolescent brain and age-related behavioral manifestations. Neurosci. Biobehav. Rev. 24, 417–463. 10.1016/S0149-7634(00)00014-2 10817843

[B68] Stolarova M. Keil A. Moratti S. (2006). Modulation of the C1 visual event-related component by conditioned stimuli: evidence for sensory plasticity in early affective perception[J]. Cerebral Cortex 16, 876–887. 10.1093/cercor/bhj031 16151178

[B69] Taylor M. Batty M. Itier R. (2004). The faces of development: a review of early face processing over childhood. J. Cogn. Neurosci. 16, 1426–1442. 10.1162/0898929042304732 15509388

[B70] Thomas K. M. Drevets W. C. Whalen P. J. Eccard C. H. Dahl R. E. Ryan N. D.. (2001). Amygdala response to facial expressions in children and adults. Biol. Psychiatry 49, 309–316. 10.1016/S0006-3223(00)01066-0 11239901

[B71] Thorpe S. Fize D. Marlot C. (1996). Speed of processing in the human visual system. Nature 381, 520–522. 10.1038/381520a0 8632824

[B72] Vaish A. Grossmann T. Woodward A. (2008). Not all emotions are created equal: the negativity bias in social-emotional development. Psychol. Bull. 134, 383. 10.1037/0033-2909.134.3.383 18444702 PMC3652533

[B73] Wang M. C. Dai X. Y. Yao S. Q. (2010). Development of Chinese big five personality inventory (CBF-PI): theoretical framework and reliability analysis. Chin. J. Clin. Psychol. 18, 545–548.

[B74] Wang X. Huang Y. Ma Q. Li N. (2012). Event-related potential P2 correlates of implicit aesthetic experience. Neuroreport 23, 862–866. 10.1097/WNR.0b013e3283587161 22922601

[B75] Wild B. Erb M. Bartels M. (2001). Are emotions contagious? Evoked emotions while viewing emotionally expressive faces: quality, quantity, time course and gender differences. Psychiatry Res. 102, 109–124. 10.1016/S0165-1781(01)00225-6 11408051

[B76] Wong T. Fung P. McAlonan G. M. Chua S. E. (2009). Spatiotemporal dipole source localization of face processing ERPs in adolescents: a preliminary study. Behav. Brain Funct. 5:16. 10.1186/1744-9081-5-16 19284600 PMC2660355

[B85] World Medical Organization (1996). Declaration of Helsinki 1964. Br. Med. J. 313, 1448–1449.

[B77] Yang J. Zeng J. Meng X. Zhu L. Yuan J. Li H.. (2013). Positive words or negative words: whose valence strength are we more sensitive to? Brain Res. 1533, 91–104 10.1016/j.brainres.2013.08.020 23958342

[B78] Yuan J. Luo Y. Yan J. H. Meng X. Yu F. Li H. (2009). Neural correlates of the females' susceptibility to negative emotions: an insight into gender−related prevalence of affective disturbances. Hum. Brain Mapp. 30, 3676–3686. 10.1002/hbm.20796 19404991 PMC6870863

[B79] Yuan J. Meng X. Yang M. Yao G. Hu L.. (2012a). The valence strength of unpleasant emotion modulates brain processing of behavioral inhibitory control: neural correlates. Biol. Psychol. 89, 240–251 10.1016/j.biopsycho.2011.10.015 22056697

[B80] Yuan J. Xu S. Yang J. Liu Q. Chen A. Zhu L.. (2011). Pleasant mood intensifies rain processing of cognitive control: ERP correlates. Biol. Psychol. 87, 17–24. 10.1016/j.biopsycho.2011.01.004 21315134

[B81] Yuan J. Zhang J. Zhou X. (2012b). Neural mechanisms underlying the higher levels of subjective well-being in extraverts: pleasant bias and unpleasant resistance. Cogn. Affect. Behav. Neurosci. 12, 175–192. 10.3758/s13415-011-0064-8 21987094

[B82] Yuan J. Zhang Q. Chen A. Li H. Wang Q.. (2007). Are we sensitive to valence differences in emotionally negative stimuli? Electrophysiological evidence from an ERP study. Neuropsychologia 45, 2764–2771. 10.1016/j.neuropsychologia.2007.04.018 17548095

[B83] Yurgelun-Todd D. A. Killgore W. D. (2006). Fear-related activity in the prefrontal cortex increases with age during adolescence: a preliminary fMRI study. Neurosci. Lett. 406, 194–199. 10.1016/j.neulet.2006.07.046 16942837

[B84] Zhu L. Chen P. J. (2012). Verification of the self-reported pubertal development scale (Chinese version). Chin. J. Sports Med. 31, 512–516.

