# Supplementary material for: Enhanced brain susceptibility to negative stimuli in adolescents: ERP evidences
Source: Front Behav Neurosci. 2015 Apr 28;9:98. doi: 10.3389/fnbeh.2015.00098 (PMC4412063; doi:10.3389/fnbeh.2015.00098)
Supplement: Supplementary file 1 [file Presentation1.PDF]

Supplementary Material 1

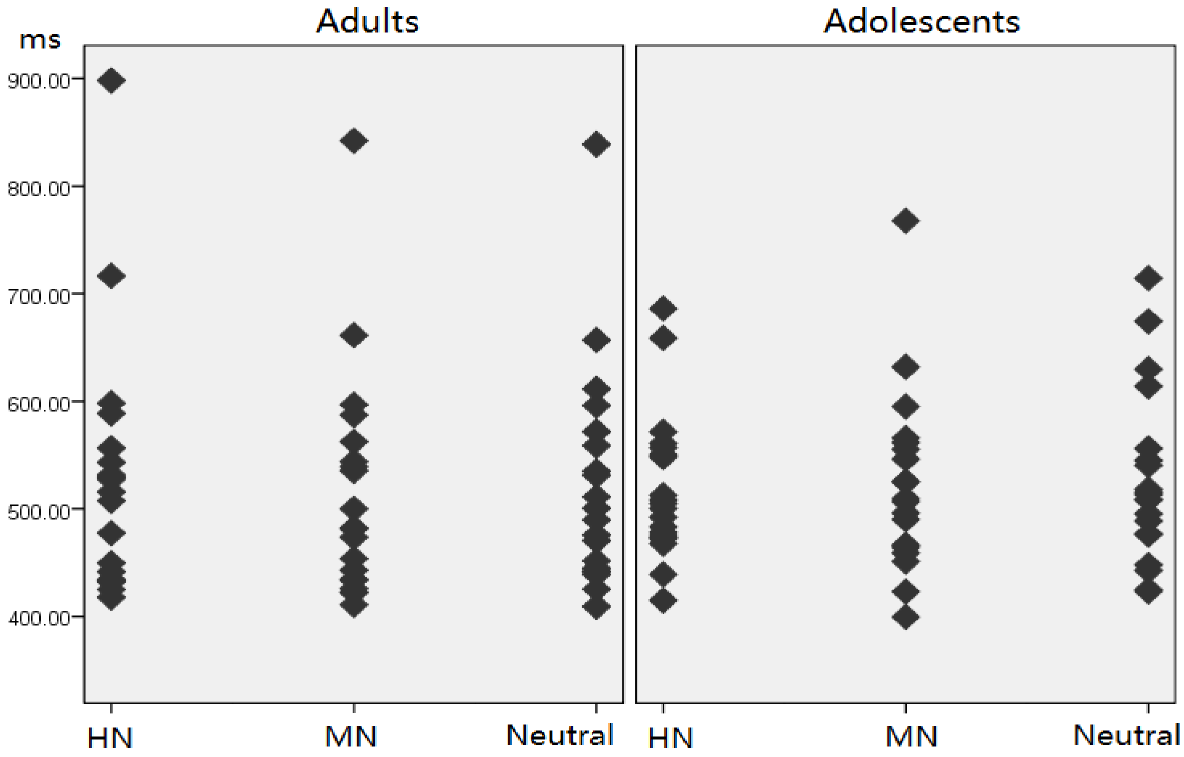

Fig.1. The scatterplot for the trimmed mean RT data per condition for each subject.

Supplementary Material 2

To further verify whether our findings of faster emotion effects for adolescents relative to adults by peak analysis is reliable, we also used a continuous approach that measured and analyzed the mean amplitudes of each 50ms epoch from stimulus onset to 500ms post stimulus. This time window covers all the components detected in the current study. Bonferroni method was used for post hoc multiple comparisons. We focused on detecting the emotion by age interaction and the main effect of emotion. Detecting an interaction in a specific window means adolescents and adults show different emotional reactions to negative stimuli in this window. Otherwise, if there is no significant emotion by age interaction and also no main effect of emotion, this means no emotion effect and emotion-related group differences in this window. If there is a significant main effect of emotion but no significant interaction with age in a specific window, this means there is a significant emotion effect regardless of age groups in this window. Based on the

functional significance and the scalp features of the ERPs in each time epoch, the 0-50ms and the 50-100ms amplitudes were analyzed at the parietal-occipital region (for early visual processing), the 100-300ms epochs in the central to frontal regions (corresponding to perceptual and attention mechanisms), and 300-500 epochs in the frontal, central and parietal regions (late cognitive processing involves p3).

The results showed a significant age by emotion interaction just in the 100-150ms, 150-200ms and the 200-250ms. In the time windows earlier than 100ms, there was neither significant emotion effect nor emotion by age interaction, consistent with our findings reported by C1 analysis. In time windows later than 250ms, no significant age by emotion interaction was detected but there was a highly significant main effect of emotion. These data further supported the results detected by peak amplitude analysis reported in the manuscript.

Supplmentary Materials

Table 1: The F and P values for the main effect of emotion(MOE) and age by emotion interaction (A&E) on the mean amplitudes of each 50ms time interval from stimulus onset to 500ms post stimulus

		0-50	50-100	100-150	150-200	200-250	250-300	300-350	350-400	400-450	450-500 (ms)
MOE	F-value	1.13	1.62	1.17	7.92	16.49	10.32	3.88	4.77	6.25	8.46
	p-value	0.33	0.20	0.23	0.001**	0.00**	0.00**	0.026*	0.013*	0.005**	0.001**
A & E	F-value	1.36	1.25	10.25	4.28	3.11	3.05	2.02	0.73	0.19	0.36
	p-value	0.26	0.29	0.00*	0.018*	0.05*	0.053	0.14	0.48	0.79	0.69

*p<0.05; **p<0.01

Table 2: The breakdown of the emotion by age interaction by testing the significance of the emotion effect (negative-neutral differences) in either sample

		Adolescents			Adults		
		100-150	150-200	200-250	100-150	150-200	200-250 (ms)
HN	MD	-1.42μV	-3.09μV	-3.53μV	1.18μV	-0.62μV	-1.29μV
	Sig.	0.039*	0.002**	0.00**	0.06	0.31	0.055
MN	MD	-2.05μV	-1.61μV	-0.92μV	1.01μV	0.98μV	0.14μV
	Sig.	0.006**	0.042*	0.32	0.11	0.24	0.77

MD: Mean Differences *p<0.05; **p<0.01
